# Author Response to Comments on “Rolling epidemic of Legionnaires’ disease outbreaks in small geographic areas”

**DOI:** 10.1038/s41426-018-0150-x

**Published:** 2018-09-05

**Authors:** C. Raina MacIntyre, Amalie Dyda, Chau Minh Bui, Abrar Ahmad Chughtai

**Affiliations:** 1 School of Public Health and Community Medicine, Faculty of Medicine, University of New South Wales, Sydney, NSW 2052 Australia; 2 College of Public Service & Community Solutions, Arizona State University, Phoenix, AZ 85004 USA

Dear Editor,

In response to the letter of Greene et al. about our study^1^, we agree that public sources including health department websites and news reports may be biased because of incomplete or censored information related to outbreaks. However, such information should be and often is openly available, and disease control efforts can benefit from critical review and different perspectives about such information. Internet-based sources are increasingly being used in public health for rapid epidemic surveillance, monitoring and analysing outbreaks^2^. Greene et al. are concerned that we did not cite two relevant published studies^3,4^. We agree these are important studies, however, they were not published at the time our paper was submitted to *Emerging Microbes and Infections* in September 2017.

At the time of submission, we had used available information from one publication^5^ and data from New York City (NYC) Health Department Website and other news sources. We did acknowledge the limitations of using publicly available data on page 8, last para, “*A limitation of this study was that most information on LD cases and epidemiological investigations were taken from public reports and news items, rather than official health department records. There is a possibility that some public information may be incorrect or incomplete*.” The key observation of our analysis is that outbreaks clustered in time and geography are rare, and missing information about cases of disease would not negate this.

Greene et al. raise both the possibility of missing data as well as the opposite scenario of more complete case ascertainment in NYC. We agree that excellent surveillance and processes as described by Greene et al. make LD outbreaks in NYC are likely more completely detected and reported than in other jurisdictions. However, we did not compare jurisdictions nor total case counts. We described geographic and temporal clustering of LD outbreaks in New York City (NYC) and Sydney (what we termed “rolling outbreaks” to indicate clustered simultaneous or serial outbreaks), which are in fact rare based on available public data^6^.

Our study did not claim the outbreaks were linked, and in fact that is precisely what is of interest and concern about them. We said that, “*While multiple outbreaks linked to a single source but occurring serially at different times have been described, rolling outbreaks associated with different exposures clustered in time and space have not been commonly reported in the past*”. For NYC outbreaks we stated that, “*The Bronx, the Opera House Hotel and Morris Park outbreaks were linked to cooling towers, but the Melrose outbreak was linked to the water supply. Interestingly, the two cooling tower outbreaks were not related to each other despite being in close proximity*”. Please see page 7, para 1. We described the unusual phenomenon of multiple different outbreaks of Legionella within a small geographic area and within a short timeframe in NYC and Sydney. We point out that while a single source (such as a hospital) can be associated with serial outbreaks of LD over many years, the phenomenon of multiple unrelated outbreaks within a small area and short timeframe is rare based on published studies and publicly available data.

**Fig. 1:**
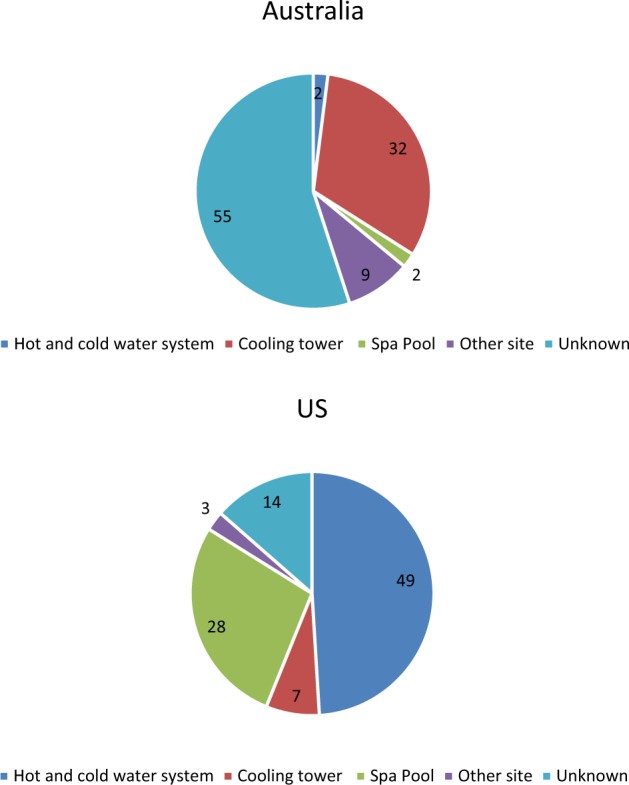
Distribution of legionella outbreak sources, USA (2000-2012) and Australia (2000–2014)

We agree with the Green et al. that the source of Legionella could be complex building water systems (i.e., within the building), with no linkage to the municipal water distribution system. However, the key point we make is the very different pattern of the source of legionella outbreaks in Australia and the US, overall. This is illustrated in Fig. 1 below, which shows that drinking water is the most common source of LD in the US (shown, based on US CDC data from 2000 to 2012), while cooling towers are the most common source of LD in Australia (Fig. 1). Similar proportions are found for New York State based on publicly available data. Regarding potable water, we did not make any assertion about the exact point from reservoir to tap in the drinking water supply that may have resulted in contamination, but make a more general comparison of the source of outbreaks at the broad level of cooling towers vs drinking water. The following definition of drinking water has been used in the CDC reports, “*Drinking water, also called potable water, is water for human consumption (e.g., drinking, bathing, showering, hand washing, teeth brushing, food preparation, dishwashing, maintaining oral hygiene) and includes water collected, treated, stored or distributed in public and individual water systems, as well as bottled wate*r”^7–11^.

We confirm that the Fig. 4 caption in the main paper is correct, but the text is incorrect and should be read as New York State. These data were collected in the most detailed available form from *MMWR Weekly Reports* published by the US Centers for Disease Control and Prevention (CDC)^7–11^. Regarding contamination of multiple cooling towers, the majority of publicly reported LD outbreaks are due to single-cooling towers, and fewer are associated with multiple towers. The largest number of towers we identified in previous published outbreaks is seven^12^, but most cases that involved multiple contaminated towers reported fewer towers than seven, often only involving two towers^13,14^. In this context, the reported number of contaminated towers in the Bronx outbreak appears unusual. We do not have an explanation for this observation, nor for the clustering of outbreaks, but discussed environmental changes such as climate for completeness, rather than making any definitive statement. A single unusually hot season, for example, is different from decades of rising mean temperatures, and it is the latter which we speculate is unlikely to explain a relatively recent phenomenon of multiple clustered outbreaks of legionella.

In summary, we respect and acknowledge the unique knowledge and expertise of Greene et al. about their own jurisdiction. However, analysis of outbreak patterns, especially across different countries, is of value despite the stated limitations, as it adds further critical analysis and insight into unusual features of outbreaks. It is important that outbreak data are publicly available in as complete a form as possible, as communicable diseases are a global concern in our connected world. The availability of such data, including surveillance data published by disease control agencies and genetic sequence data, for example, in GeneBank, are widely used for research and analysis for public health and disease control, despite being incomplete and subject to biases. Other initiatives which recognise the value of open analysis of epidemics include HealthMap and Promed Mail^15,16^. Critical analysis of such data by a wide range of stakeholders can only benefit disease control efforts, and would be improved by mechanisms to enhance the completeness of such data. While the WHO does make data available for some outbreaks, these data usually do not contain enough detail for analysis. A public, searchable repository of outbreak data from health agencies around the world may improve global disease control.
